# The Relationship Between COVID 19 Anxiety and Dementia Caregivers Burden and Suffering

**DOI:** 10.1093/geroni/igab046.3747

**Published:** 2021-12-17

**Authors:** Kaitlyn Lucas, Eleanor Batista-Malat, Seho Park, Shelly Johns, Nicole Fowler, Katherine Judge

**Affiliations:** 1 Cleveland State University, Westlake, Ohio, United States; 2 Indiana University School of Medicine, Regenstrief Institute, Indiana, United States; 3 Indiana University School of Medicine, Department of Biostatistics, School of Medicine, Indiana, United States; 4 Indiana University School of Medicine, Center for Health Services Research, Indianapolis, Indiana, United States; 5 Indiana University School of Medicine, IU Center for Aging Research, Indianapolis, Indiana, United States; 6 College of Sciences and Health Professions, Cleveland State University, College of Sciences and Health Professions, Ohio, United States

## Abstract

The impact of COVID-19 on dementia caregivers is gaining new interest. It is unknown how the pandemic has impacted caregivers’ burden and existential suffering. Analyses were performed on data for dementia caregivers (n=89) enrolled in the Indiana University Telephone Acceptance and Commitment Therapy for Caregivers (TACTICs) pilot trials. Individuals were primary caregivers of a family member with dementia and had clinically significant anxiety measured by a GAD-7 score >10 or between 5-9 with reported interference in life. COVID-19 anxiety was measured using the NIH CoRonavIruS Health Impact Survey (CRISIS) questions. Caregivers were on average 55.2 years of age with 56.2% being child or child-in-law, 71.9% were white and 24.7% were Black. Mean burden scores, measured by the Zarit Burden Index, were higher (44.29) compared to means reported across the literature (26.7) indicating the sample experienced higher than normal levels of burden. Mean existential suffering scores measured by the subscale of Experience of Suffering Scale were lower (9.37) compared to means across the literature (11.5) indicating that overall participants experienced lower levels of existential suffering compared to those in previous studies. A significant relationship was found between COVID-19 anxiety and burden levels (x2= 9.07, p<0.05), with higher levels of COVID-19 anxiety associated with greater burden. A non-significant relationship was found between COVID-19 anxiety and existential suffering (x2=5.99, p=0.11). Results highlight the impact of COVID-19 anxiety as an external stressor on dementia caregiving. and the importance of considering context of external stressors when implementing intervention protocols for caregivers of individuals with dementia.

